# Features and development trends of primary care research conducted by practice-based research networks from 1991 to 2023: a scoping review protocol

**DOI:** 10.1186/s13643-023-02395-y

**Published:** 2023-12-13

**Authors:** Yang Wang, Xinyang Cao, Zhijie Xu, Hai Fang

**Affiliations:** 1 School of Public Health, Peking University, Beijing, 100191 China; 2 China Center for Health Development Studies, Peking University, Beijing, China; 3 Chinese General Practice Publishing House, Co., Ltd, Handan, China; 4 Department of General Practice, The Second Affiliated Hospital, Zhejiang University School of Medicine, Hangzhou, China

**Keywords:** Primary care, Primary health care, General practice, Family medicine, Practice-based research networks

## Abstract

**Background:**

Practice-based research networks (PBRNs) have been recognized as essential laboratories and mechanisms for developing primary care research. This scoping review aims to examine and map the features and development trends of productivity, research categories, and methods in original primary care research conducted by global PBRNs between 1991 and 2023.

**Methods:**

We have assembled an interdisciplinary team that will undertake this scoping review, following the framework developed by Arksey and O’Malley. Targeted literature includes original primary care research conducted by PBRNs, published from January 1, 1991, to December 31, 2023. An integrated search strategy will gather publications from 3 electronic databases (PubMed, Web of Science, and Embase), 16 major primary health care journals, and 364 relevant organizations. Two experienced researchers will independently screen the titles, keywords, and abstracts of all references and extract data regarding eight key elements. Disagreements between the reviewers will be resolved through group discussions, moderated by a third reviewer. Articles to be included will (1) be conducted in the primary care context, (2) be led by PBRNs, (3) provide a full report of original research, and (4) be published in a peer-reviewed journal between the aforementioned dates in any language. Exclusions encompass reviews, letters, commentaries, case reports, and conference papers. Final data will be displayed using tables and charts according to different conceptual categories.

**Discussion:**

This scoping review is one of the initial attempts to delineate the development trends and features of primary care research conducted by PBRNs. This study will provide reference information for researchers in countries/regions that are building their research infrastructure and capacity in general practice, family medicine, and primary care.

**Systematic review registration:**

Registered in OSF on July 25, 2022 (https://osf.io/zgv9c).

**Supplementary Information:**

The online version contains supplementary material available at 10.1186/s13643-023-02395-y.

## Background

Enhancing primary health care (PHC) is the most crucial approach to improving people’s health and social well-being and achieving health-related sustainable development goals [1]. In the 14 key levers supporting the successful implementation of PHC proposed by the World Health Organization (WHO), the role of developing PHC-oriented research is to support all the other levers by creating and providing knowledge, evidence, and intelligence. Therefore, it has unique features: covering all components of PHC, cooperating with a broad range of stakeholders, and pragmatically addressing important issues for the local community (“Act local, think global”) [2, 3].

Primary care research is defined as “research done in a primary care context” [4] and involves five research types (basic research, clinical research, health service research, health system research, and educational research) [5]. It is a “bottom-up” multidisciplinary research area that views primary care facilities and communities as the fundamental units and places the research focus on them [6]. The collaboration between primary care practitioners and researchers is crucial for developing primary care research, as this can integrate science and practice and facilitate mutual reinforcement [7, 8].

Over the last half-century, a key approach to achieving this collaboration has been practice-based research networks (PBRNs) [8]. The Agency for Healthcare Research and Quality (AHRQ) defines this as “a group of ambulatory practices devoted principally to the primary care of patients and affiliated in their mission to investigate questions related to community-based practice and to improve the quality of primary care” [9]. It highlights PBRNs’ three core features as a research laboratory: engagement in primary care research based on clear and stable purpose, structure, and resources [10, 11]; components including multiple primary care practices favorable to data collection [12]; and a focus on answering research questions derived from practice or conducting research, which is important for practice, especially regarding translating evidence into practice [13]. In some countries and regions, it is also named “primary care research network” [12].

The value of PBRNs for developing primary care research and related health science research has been widely recognized by the global academic community in recent decades [12, 14–16]. Since the initiation of health system reforms in 2009, which focused on augmenting human and financial resources for primary care, China has witnessed a rapid expansion of primary care and general practice [17]. Against this backdrop, a common concern in the local academic community in recent years is how to cultivate primary care research in China, a middle- to low-income country with a distinct health system backdrop, in order to better foster local primary care practices [18]. Consequently, creating a comprehensive map that chronicles the historical development trends in this domain over the past 30 years would serve as a macro-level reference for health and research policymakers and administrators, both in China and in countries with similar circumstances. Such a reference would provide them a panoramic view of the distinctive history and evolution of research derived from PBRNs.

In our previous study, we found that the number of studies recorded in the AHRQ’s literature database of PBRNs has been increasing rapidly since the 1990s [19]. The number of registered PBRNs and published primary care studies also increased during the same period [8, 16, 20, 21]. The spread of registered PBRNs across the world may be reflective of the change in the productivity of PBRNs. This trend is also seen in countries outside of North America, such as the UK, the Netherlands, and Australia [22, 23]. In addition, some changes to primary care research methods during this period may also reflect the evolution in the features of the studies conducted by PBRNs [24–27]. Therefore, we would like to further examine and map the global features and development trends of productivity, research categories, and methods in original primary care research conducted by PBRNs between 1991 and 2020. In bibliometrics, productivity is usually approximate to the number of available scientific publications, which is a key indicator for measuring the research output of a researcher, institution, or region/country [28], and has been used to assess the development of primary care research [20, 21].

We identified the innovative value of our study after conducting a preliminary search in PubMed, Web of Science, Embase, Cochrane Database, and Open Science Framework.

## Methods

### Study design

We plan to conduct this work following the recommended procedures developed by Arksey and O’Malley [29]. The procedure is a common and established approach for designing and conducting a scoping review and involves five stages: (1) identifying the research question, (2) identifying relevant studies, (3) selecting studies, (4) charting the data, and (5) collating, summarizing, and reporting results. Therefore, we assembled an interdisciplinary team (including YW and HF, primary health care researchers; XC, scientific editors of a primary care academic journal; and ZX, an academic general practitioner) to conduct this scoping review. Furthermore, we will report our results according to the PRISMA Extension for Scoping Reviews (PRISMA-ScR) checklist [30]. The scoping review protocol is being registered in the Open Science Framework database (https://osf.io/zgv9c).

### Stage 1: Identifying the research question

This study aims to explore the following research question: “What are the features and development trends of productivity, research type, and methods of original primary care research that were conducted by PBRNs and published between 1991 and 2023?” We plan to initiate our search in January 2024. In this work, we restricted the meaning of “primary care research” to “research done in a primary care context,” based on Starfield’s definition [4]. As the meaning of “primary care context” may vary in different countries and regions, we will refer to the reference book on global primary care research published by the World Organization of Family Doctors (WONCA), which describes the primary care context in different regions [31]. In addition, we will also refer to the definitions proposed by major local academic associations. For example, in the USA, the American Academy of Family Physicians defines “primary care” as “health care services by physicians and their health care teams” and limited “primary care physician” to “a specialist in family medicine, general internal medicine, or general pediatrics” [32]. Further, according to the AHRQ definition, we defined “research conducted by PBRNs” as articles that meet any of the following conditions: (1) clearly labeled as a study by PBRNs; (2) the first author is affiliated to PBRNs (that is, PBRN researchers must have made major contributions); and (3) the contribution of PBRNs have been reported in the papers’ introduction or methods sections [9].

### Stage 2: Identifying relevant studies

In our previous study, we recognized that adding more databases to the three main databases (PubMed, Web of Science, and Embase) does not improve the efficiency of obtaining target publications [19]. Moreover, the accuracy of the papers collected in AHRQ’s PBRN literature database that were defined as research conducted by PBRNs was less than 50% [19]. Therefore, we plan to search the target publications through three different approaches and then combine them.

#### Electronic database

We will conduct a comprehensive search of original articles published from January 1, 1991, to December 31, 2020, in PubMed, Web of Science, and Embase through a search strategy developed in previous work [33, 34] (Supplement Table 1).

#### Hand-searching of key journals

We will manually search 16 major primary health care journals indexed in the Journal Citation Report. Based on our initial search, it is possible to find published original research conducted by PBRNs in them (Supplement Table 2).

#### Relevant organizations

We will search PubMed for each PBRN’s name as an affiliation according to a PBRNs list (Supplement Table 3) that we compiled—which was based on the PBRNs mentioned in three important PBRNs-related information sources [6, 35, 36]—and three studies conducted by researchers experienced with PBRNs [19, 34, 37]. We reviewed the original list and removed PBRNs that did not meet our research aim; for example, we removed all non-primary care PBRNs from AHRQ’s PBRN registry website.

### Stage 3: Selecting studies

After the search, all the information regarding the included articles will be imported to EndNote X9.2 software (Clarivate Analytics, Philadelphia, USA, 2019). We will remove duplicates using the software functions. The revision and study selection will then be performed by importing the literature information into Rayyan, a web and mobile app for conducting systematic reviews that improves screening efficiency with its semi-automation function [38]. Two researchers with expertise in reviewing academic papers (YW, XC) will screen the titles, keywords, and abstracts of the first 300 references to improve the eligibility criteria and ascertain that they are consistently understood through weekly comparisons and group discussions. A follow-up screening of approximately 10,000–15,000 articles will begin when the consistency between the two reviewers is greater than 90%. If the two reviewers fail to reach a consensus, the third researcher (ZX) will moderate specific group discussions to resolve discrepancies. Finally, we will use a PRISMA flow diagram to demonstrate the study selection process. Articles that meet the following criteria will be included:

1. Conducted in the primary care context
2. Conducted by PBRNs, including the three cases we elaborated on in Stage 1
3. A full report of original research
4. Published in a peer-reviewed journal between January 1, 1991, and December 31, 2023
5. Published in any language

We will exclude reviews, letters, commentary, case reports, and conference papers. Given that the main mechanism of PBRNs in supporting primary care research is to generate research from primary care practice and translate or implement evidence into practice, we will include only original research in this review and will exclude non-research papers as well as reviews. We excluded review papers because the fundamental purpose of review studies is to “gather research, getting rid of rubbish and summarizing the best of what remains” [39], which essentially makes them secondary and information studies that can be conducted by any type of institution. Thus, they are not closely related to the unique mechanism between PBRNs and primary care research.

### Stage 4: Charting the data

We plan to extract nine elements from each article according to the proposed data extraction form (Table 1). Of these elements, six (article title, publication year, journal’s name, first authors’ name, first authors’ affiliation, and country/region) can be extracted from the bibliometric record. We will clean and classify them with the support of Openrefine, an AI-based software that supports the efficient cleaning and transformation of bibliometric data [40]. In addition, two reviewers (YW and XC) will independently classify each included article by research category and research method according to the taxonomy of primary care research developed by WONCA and the research methods list summarized from the “appropriate research methodology” section of Research Agenda for General Practice/Family Medicine and Primary Health Care in Europe [3]. They may also add new but relevant classifications based on subsequent findings outside the aforementioned frameworks. They will first classify 50 papers, and after the raw agreement is higher than 90%, they will classify the remaining papers.

**Table 1:** Proposed data extraction form

Article ID:	
Article title:	
Publication year:	
Journal name:	
First author’s name	
PBRN’s name	
Country/region	PBRNs’ country or region (e.g., USA, UK, Canada, multinational)
Type of first authors’ affiliation	Select from the following categories: (1) Department of family medicine/general practice/primary care/community health; (2) Other departments in the university; (3) Academies; (4) Hospitals, clinics, or local health networks; (5) PBRNs; (6) Research institutes; (7) Companies; (8) Government branch; (9) Foundation; (10) Others
Research category:	Select from the following categories: (1) Basic research; (2) Clinical research; (3) Health services research; (4) Health systems research; (5) Educational research; (6) Other types
Research method:	Select from the following categories: (1) Cross-sectional study; (2) Prospective longitudinal study; (3) Retrospective longitudinal study; (4) Interventional study (excluding randomized controlled trials); (5) Randomized controlled trial; (6) Qualitative study; (7) Mixed methods research; (8) Other methods (e.g., questionnaire development study, implementation science, quality improvement study)

### Stage 5: Collating, summarizing, and reporting results

The data will be presented in tables and charts depending on the conceptual categories. Tables and charts will present the distribution of years, countries/regions, type of first authors’ affiliations, research categories, and methods of the identified studies. Narrative summaries will be used to provide additional explanatory information based on the collected data. We also plan to use descriptive analysis and comparative statistics (including chi-squared test and nonparametric tests) to find possible common features and the development trend of a group of studies during a stable period.

## Discussion

Based on our knowledge, this scoping review is one of the initial attempts to delineate the development trends and features of primary care research conducted by PBRNs. In it, we will generate a systematic search strategy to combine the information obtained from multiple approaches to ensure the comprehensiveness of the search. This study will provide reference information for researchers in countries/regions that are building their research infrastructure and capacity in general practice, family medicine, and primary care.

The findings of this study will present a macroscopic outline of how pioneer researchers in primary care, within various national and regional research environments and institutions, choose distinct principal research domains and methodologies, utilizing PBRNs as a fundamental base to conduct primary care research work. By offering a holistic view of the PBRN-grounded primary care research landscape across diverse regions and research settings, our work aspires to identify and illuminate the varied strategies, challenges, and successes encountered by researchers globally. Thus, this review not only encapsulates the historical and current trajectories of primary care research but also aims to equip researchers, practitioners, and policymakers with insights and benchmarks that can inform the design, initiation, and optimization of future PBRN studies and initiatives in different sociocultural and institutional contexts.

As the main contribution of PBRNs to primary care research is generating practice-based knowledge, this study will include only original articles and exclude reviews. This scoping review may miss some studies published in non-English journals that are not indexed in the Journal Citation Report.

### Supplementary Information

**Additional file 1: Table S1. **Search strategy for PubMed, WOS, and Embase.** Table S2. **List of 16 primary care and family medicine journals.** Table 3. **List of PBRNs.

## Data Availability

Not applicable.

## Abbreviations

PBRNs: Practice-based research networks
PHC: Primary health care
WHO: World Health Organization
AHRQ: Agency for Healthcare Research and Quality
PRISMA-ScR: PRISMA Extension for Scoping Reviews
WONCA: World Organization of Family Doctors
