# Supplementary material for: Features and development trends of primary care research conducted by practice-based research networks from 1991 to 2023: a scoping review protocol
Source: Syst Rev. 2023 Dec 13;12:229. doi: 10.1186/s13643-023-02395-y (PMC10717402; doi:10.1186/s13643-023-02395-y)
Supplement: Supplementary file 1 — Additional file 1: Table S1. Search strategy for PubMed, WOS, and Embase. Table S2. List of 16 primary care and family medicine journals. Table 3. List of PBRNs. [file 13643_2023_2395_MOESM1_ESM.docx]

**Supplement table 1. Search strategy for PubMed, WOS, and Embase**

| PubMed  (13418 Results) | (Clinic*[tiab] OR practi*[tiab] OR primary[tiab] OR physician*[tiab] OR refer*[tiab] OR visit*[tiab] OR outpatient*[tiab] OR consult*[tiab] OR family[tiab] OR communit*[tiab] OR ambulatory[tiab] OR centre*[tiab] OR centre*[tiab] OR office[tiab])  AND  ("research network*"[All Fields] OR "pbrn*"[All Fields] OR "pbrn"[Affiliation] OR "research network"[Affiliation] OR "practice network"[Affiliation] OR "ambulatory network"[Affiliation] OR "research collaborative"[Affiliation] OR "research consortium"[Affiliation] OR "community health network"[Affiliation] OR "care network"[Affiliation]) |
| --- | --- |
| WOS  (4525 Results) | (TS = (primary care OR primary health OR family physician* OR general practi* OR family practi* or outpatient* or clinic* or ambulatory or health centre* or health centre* or office))  AND  ((TS = ("research network*" OR “PBRN*”)) OR (OO = ("research network" OR "practice network" OR "ambulatory network" OR "research collaborative" OR "research consortium" OR "community health network" OR "care network"))) |
| Embase  (14363 Results) | (family OR physician* OR practice*:de,it,lnk,ab,ti OR 'primary care' OR 'primary health care'/exp OR primary:de,it,lnk,ab,ti OR (general NEXT/1 pract*) OR gp:ab,ti OR gps:ab,ti)  and  ('research network*':ti,ab,kw OR 'pbrn*':ti,ab,kw) OR ('research network':ff OR 'practice network':ff OR 'ambulatory network':ff OR 'research collaborative':ff OR 'research consortium':ff OR 'community health network':ff OR 'care network':ff) |

**Supplement table 2. List of 16 primary care and family medicine journals**

| Title | Publication Start Year | Related Title | Country of Publication |
| --- | --- | --- | --- |
| **Journal of the American Board of Family Medicine** | 1988 | Journal of the American Board of Family Practice | USA |
| **Journal of family practice** | 1974 |  | USA |
| **Annals of family medicine** | 2003 |  | USA |
| **Family practice** | 1984 |  | UK |
| **BMC family practice** | 2000 | BMC primary care | UK |
| **Canadian family physician** | 1967 | Médecin de famille canadien | Canada |
| **British journal of general practice** | 1990 |  | UK |
| **European journal of general practice** | 1995 |  | Netherland |
| **Atencion primaria** | 1983 |  | Spain |
| **Semergen** | 1996 | Sociedad Española de Medicina Rural y Generalista | Spain |
| **South African Family Practice** | 1994 |  | South Africa |
| **Korean Journal of Family Medicine** | 2009 | Kajŏng Ŭihakhoe chi | Korea |
| **Australian journal of general practice** | 2018 | Australian family physician | Australia |
| **African Journal of Primary Health Care and Family Medicine** | 2009 |  | South Africa |
| **Scandinavian Journal of Primary Health Care** | 1983 |  | Sweden |
| **Journal of primary health care** | 2009 | New Zealand family physician | Australia |

\

**Supplement table 3. List of PBRNs**

| **Name** | **Acronym** | **Country** |
| --- | --- | --- |
| General Practice Research Network | GPRN | Australia |
| Australian Sentinel Practice Research Network | ASPREN | Australia |
| Electronic Practice Based Research Network | e-PBRN | Australia |
| Victorian Primary Care Practice-Based Research Network | VicReN | Australia |
| Illawarra and Southern Practice Research Network | ISPRN | Australia |
| Primary Healthcare Research Network-General Practice | PHReNet-GP | Australia |
| South Australian Research Network | SARNet | Australia |
| University Family Practice Network | UFPN | Australia |
| University of Adelaide PBRN |  | Australia |
| Hunter New England Central Coast Network of Research General Practices | NRGP | Australia |
| Monash University practice-based research network |  | Australia |
| Australian Primary Care Research Network |  | Australia |
| Research Excellence in Aboriginal Community Controlled Health | REACCH | Australia |
| The research network of the Department of General Practice at the Medical University of Vienna |  | Austria |
| Intego |  | Belgium |
| Belgian Network of Sentinel General Practices |  | Belgium |
| ResoPrim Project |  | Belgium |
| Canadian Primary Care Sentinel Surveillance Network | CPCSSN | Canada |
| TARGet Kids Toronto Area Research Group |  | Canada |
| Centre for Studies in Primary Care | CSPC | Canada |
| University of Toronto Practice-Based Research Network | UTOPIAN | Canada |
| Manitoba Primary Care Research Network |  | Canada |
| Thames Valley Family Practice Research Unit | TVFPRU | Canada |
| North Toronto Primary Care Research Network | NORTREN | Canada |
| Southern Alberta Primary Care Research Network | SAPCReN | Canada |
| WestView Primary Care Network | WPCN | Canada |
| Northern Alberta Primary Care Research Network | NAPCReN | Canada |
| McMaster University Sentinel and Information Collaboration | MUSIC | Canada |
| Canadian Primary Health Care Research and Innovation Network | CPHCRIN | Canada |
| Deliver Primary Healthcare Information project | DELPHI | Canada |
| Maritime Family Practice Research Network | MaRNet‑FP | Canada |
| Practice-Based Research Network, Université de Sherbrooke |  | Canada |
| BC Primary Health Care Research Network | BC-PHCRN | Canada |
| Kingston Practice-based Research Network |  | Canada |
| McGill Practice-Based Research Network |  | Canada |
| Réseau de recherche axé sur les pratiques de première ligne de l'Université Laval | Université Laval PBRN | Canada |
| Réseau de recherche en soins primaires de l'Université de Montréal | RRSPUM | Canada |
| Eastern Ontario Network |  | Canada |
| Knowledge Network in Integrated Primary Care Health Services / Réseau de Connaissances en Services et Soins de Santé Intégrés de Première Ligne au Québec | Knowledge-1 Quebec / Réseau-1 Québec | Canada |
| Quebec Practice-Based Research Network of Laval University / Réseau de recherche axé sur les pratiques en première ligne Universite Laval | QPBRN / RRAPPL | Canada |
| University of Western Ontario PBRN |  | Canada |
| Tutka Primary Care Research Network | TUTKA | Finland |
| Loire-Atlantic department using a general practice research network | Resomed 44 | France |
| Strasbourg General Medicine Department practice based research network | GMD PBRN | France |
| Sentinelles network |  | France |
| Research Practice Network Baden-Württemberg | FoPraNet-BW | Germany |
| CONTinuous morbidity registration Epidemiologic NeTwork | CONTENT | Germany |
| Bavarian research network for general practice | BayFoNet | Germany |
| DESAM-ForNet |  | Germany |
| Hannover Medical School started to build a Practice Based Research Network | | Germany |
| North-Rhine Westphalian General Practice Research Network | NRW-GPRN | Germany |
| Practice-Based Research Networks Dresden/Frankfurt am Main | SaxoForN | Germany |
| Research Practice Network East | RESPoNsE | Germany |
| Research Practices Halle-Leipzig | RaPHaeL | Germany |
| Cretan Rural Practice-based Research Network |  | Greece |
| Holistic Healthcare and Research Centre | Holistic Healthcare and Research Centre | India |
| South Asian PBRN | SAIHP PBRN | India |
| European General Practice Research Network | EGPRN | International |
| GRACE project(study) group | GRACE-01 | International |
| European Academy of Paediatrics Research in Ambulatory Setting network | EAPRASnet | International |
| European Sentinel Network of the GPs to Monitor End- of-Life Care | EURO SEN TI-MEL C | International |
| Irish Primary Care Research Network | IPCRN | Ireland |
| University of Limerick Education and Research Network for General Practice | ULEARN-GP | Ireland |
| Western Research and Education Network | WestREN | Ireland |
| West of Ireland Research and Education Network | WestREN | Ireland |
| Israeli Family Medicine(Practice) Research Network | RAMBAM | Israeli |
| Centro Studi e Ricerca in Medicina Generale | CSeRMeG PBRN | Italy |
| Japan Association for Development of Community Medicine Practice Based Research Network | JADECOM-PBRN | Japan |
| Primary care practice-based research network in Japan |  | Japan |
| Centre for Family Medicine Development Practice Based Research Network | CFMD-PBRN | Japan |
| Tottori University Primary Care Research Network | Tori Net | Japan |
| Pohang . Gyeongju Primary Care Research Network |  | Korea |
| Royal New Zealand College of General Practitioners | RNZCGP CRN | New Zealand |
| General Practice Information Network |  | New Zealand |
| Primary Care Research Network | PCRN | Poland or Norway |
| Pacific People's Health Advisory Group PBRN | PPHAG | Samoa, Tonga, Cook Islands, Niue, and New Zealand |
| A Sign Health Polyclinics Initiative for Research Excellence | ASPIRE | Singapore |
| African Sentinel Practitioner Research Network | SASPREN | South Africa |
| South African Sentinel Practitioner Research Network | SASPREN | South Africa |
| The Stellenbosch University Family Physician Research Network | SUFPREN | South Africa |
| The Research Network on Preventive Activities and Health Promotion | REDIAPP | Spain |
| Spanish Preventive Services and Health Promotion Research Network |  | Spain |
| The experience from Seminars of Innovation on Primary Care | SIAP | Spain |
| Academic Primary Healthcare Network | APHN | Sweden |
| Swiss Sentinella Surveillance network |  | Switzerland |
| Family Medicine ICPC-Research Using Electronic Medical Records | FIRE Project | Switzerland |
| Swiss Primary Care Active Monitoring Network | SPAM | Switzerland |
| Continuous Morbidity Registration | CMR | The Netherlands |
| Rotterdam General Practitioners Project | ROHAPRO | The Netherlands |
| Nijmegen Family Practice Academic Network |  | The Netherlands |
| Registration Network Groningen | RNG | The Netherlands |
| Registration Network Family Practices | RNH | The Netherlands |
| Transition project | Thanshis | The Netherlands |
| Family Medicine Network | FaMe-net | The Netherlands |
| Dutch Transition Project |  | The Netherlands |
| Nijmegen Monitoring Project | NMP | The Netherlands |
| Dutch Sentinel General Practice Network | SGPN | The Netherlands |
| Utrecht General Practitioner Research Network |  | The Netherlands |
| Weekly Returns Service | WRS | UK |
| Medical Research Council General Practice Research Framework | MRC GPRF GPRF | UK |
| Trent Focus Collaborative Research Network |  | UK |
| Scottish Primary Care Research Network | SPCRN (previously SPPIRe) | UK |
| Wessex Primary Care Research Network |  | UK |
| South Thames Area Research Network | STaRNeT | UK |
| Northern Primary Care Research Network | NoRen | UK |
| West London Primary Care Research Network Community Interest Company | WeLReN | UK |
| East London and Essex Network of Researchers | ELENoR | UK |
| Health and Occupation Research network in General Practice | THOR-GP | UK |
| Keele GP Research Network |  | UK |
| Kent, Surrey and Sussex Primary Care Research Network |  | UK |
| General Practice Data Retrieval Project | GPDRP | UK |
| Greater London Primary Care Research Network |  | UK |
| North Staffordshire General Practice Research Network |  | UK |
| primary care research network in Oxfordshire |  | UK |
| UK primary care research network |  | UK |
| Cumbria Primary Care Research Network | CumbReN | UK |
| Cutting Out Needless Deaths Using Information Technology network | CONDUIT | UK |
| Dumfries and Galloway Primary Care Research Network |  | UK |
| Tayside's Primary Care Research and Development Network | Tayren | UK |
| Community Clinical Oncology Program | CCOP | USA |
| Research Involving （in）Outpatient Settings Network | RIOS Net | USA |
| Dartmouth Practice-based Research Network | The Dartmouth CO-OP Project | USA |
| Oregon Rural Practice-based Research Network | ORPRN | USA |
| Palo Alto Medical Foundation Research Institute | PAMFRI | USA |
| Clinical Directors Network, Inc. | CDN | USA |
| Care Coordination Institute | CCI | USA |
| Cincinnati Area Research Improvement Group network | CARInG Network | USA |
| OCHIN Practice-Based Research Network (formerly SafetyNet West) | OCHIN PBRN | USA |
| Pediatric Practice Research Group | PPRG | USA |
| Metropolitan Detroit Practice-based Research Network | MetroNet | USA |
| American Academy of Family Physicians National Research Network | AAFP NRN | USA |
| Shared(State) Networks of Colorado Ambulatory Practices and Partners | SNOCAP | USA |
| Ambulatory Sentinel Practice Network | ASPN | USA |
| Oklahoma Physicians Resource/Research Network, Inc. | OKPRN | USA |
| Cancer Prevention and Control Research Network | CPCRN | USA |
| Pediatric Physicians' Organization at Children's | PPOC | USA |
| Distributed Ambulatory Research in Therapeutics Network | DARTNet | USA |
| Community Health Center Network | CHCN | USA |
| Community Child Health Network | CCHN | USA |
| Practice Partner Research Network | PPRNet | USA |
| High Plains Research Network | HPRN | USA |
| Slone Center Office-based Research Network | SCOR Network | USA |
| International Primary Care Network | IPCN | USA |
| Scalable Architecture for Therapeutic Inquiries Network | SAFTINet | USA |
| Association of Asian Pacific Community Health Organizations | AAPCHO | USA |
| Iowa Research Network | IRENE | USA |
| Alliance of Chicago Community Health Services | ACCHS | USA |
| Association of Pediatric Program Directors Longitudinal Educational Assessment Research Network | APPD LEARN | USA |
| University of Missouri PBRN |  | USA |
| Upper Peninsula Research Network | UPRNet | USA |
| Military Primary Care Research Network | MPCRN | USA |
| Continuity Research Network | CORNET | USA |
| Access Community Health Network | ACHN | USA |
| ProHealth Physicians | PHP | USA |
| Accelerating Data Value Across a National Community Health Center Network | ADVANCE Clinical Data Research Network | USA |
| OneFlorida Clinical Research Consortium | OneFlorida CRC | USA |
| The National practice-based research network of the American Academy of Pediatrics | | USA |
| Pediatric Research in Office Settings research network | PROS | USA |
| Wisconsin Research and Education Network | WREN | USA |
| Center for Community Health Education Research and Service | CCHERS | USA |
| The Pediatric Research Consortium | PeRC | USA |
| Kentucky Ambulatory Network | KAN | USA |
| North Texas Primary Care Practice-based Research Network | NorTex-PBRN | USA |
| PRImary care MultiEthnic Network | PRIME Net | USA |
| South Texas Ambulatory Research Network | STARNet | USA |
| WWAMI region Practice and Research Network Washington | WPRN | USA |
| Ambulatory Network for Scholarship and Research | ANSR | USA |
| Colorado Child Outcomes Network | COCONet | USA |
| Colorado Research Network | CaReNet | USA |
| Improvement Science Research Network | ISRN | USA |
| South Florida Primary Care Practice-Based Research Network |  | USA |
| Community Based Research Network | CBRN | USA |
| Family Health Centers at NYU Langone | FHCNL | USA |
| Puget Sound Pediatric Research Network | PSPRN | USA |
| Cincinnati Pediatric Research Group | CPRG | USA |
| Duke Primary Care Research Consortium | PCRC | USA |
| South Carolina Pediatric Practice Research Network | SCPPRN | USA |
| University HealthCare Alliance | UHA | USA |
| Northeastern Ohio Network | NEON | USA |
| Women's Health Research Network | WHRN | USA |
| Harrisburg Area Research Network | HARNET | USA |
| Community Health Applied Research Network | CHARN | USA |
| Crozer-Keystone Health Network | CKHS | USA |
| Los Angeles County Department of Health Services, Ambulatory Care Network - Research & Innovation | LAC DHS ACN-R&I | USA |
| Residency Research Network of Texas | RRNeT | USA |
| Southwestern Ohio Ambulatory Research Network | SOAR-Net | USA |
| Utah Health Research Network |  | USA |
| Better Outcomes through Research for Newborns | BORN | USA |
| Residency Research Network of South Texas | RRNeST | USA |
| Ambulatory Research Community Health Network | ARCHNet | USA |
| Asthma Training To Accelerate Communication and Knowledge | ATTACK | USA |
| Greater rochester practice-based research network | GR-PBRN | USA |
| LA Net Community Health Network OR University of Southern California -Los Angeles PBRN | LA Net | USA |
| Michigan Research Network | MIRNET | USA |
| Pediatric PittNet: University of Pittsburgh CTSI PBRN | Pediatric PittNet | USA |
| Rural Primary Care Practice and Research Network | RPCPRP | USA |
| San Francisco Bay Area Collaborative Research Network | SF Bay CRN | USA |
| Southeast Regional Clinicians Network | SERCN | USA |
| Southern Primary care Urban Research Network | SPUR-Net | USA |
| Upstate New York Practice Based Research Network | UNYNET | USA |
| Alliance for Research in Community Health | ARCHNet | USA |
| Appalachian Research Network | AppNET | USA |
| Central Appalachia Inter-Professional Pain Education Collaborative |  | USA |
| Central Texas Primary Care Research Network | CenTexNet | USA |
| Cleveland Clinic Ambulatory Research Network | CleAR-eN | USA |
| Collaborative Care Research Network | CCRN | USA |
| Indianapolis Discovery Network for Dementia | IDND | USA |
| International Federation of Primary Care Research Networks | IFPCRN | USA |
| Midwestern practice-based research network |  | USA |
| Minnesota Academy of Family Physicians Research Network | MAFPRN | USA |
| National Interdisciplinary Primary Care Practice-Based Research Network | NIPC-PBRN | USA |
| New Jersey primary care research network | NJPCRN | USA |
| North Carolina Family Medicine Research Network | NC-FM-RN | USA |
| Providence Primary Care Research Network in Oregon |  | USA |
| Safety Net Provider's Strategic Alliance | SNPSA | USA |
| Texas A&M University Health Science Center A&M Rural and Community Health Institute (ARCHI Institute) | TAMUHSC-ARCHI | USA |
| Virginia Practice Support and Research Network | VaPSRN | USA |
| Washington University Pediatric and Adolescent Ambulatory Research Consortium | WU PAARC | USA |
| UCSF/Stanford Collaborative Research Network | CRN | USA |
| Ambulatory Care Research Network | ACRN | USA |
| Capital Area Primary Care Research Network | CAPRICORN | USA |
| Central Ohio Practice Based Research Network | COPBRN | USA |
| Centricity Health Care User Research Network | CHURN | USA |
| Clinicians Enhancing Child Health | CECH | USA |
| Cumberland Pediatric Foundation | CPF | USA |
| Developmental Disabilities PBRN | DD-PBRN | USA |
| Diabetes Wellness and Prevention Coalition | DWPC | USA |
| Family Practice Training Site Research Network | FPTSRN | USA |
| FM-Pittnet | FM PittNet | USA |
| Great Lakes Research Into Practice Network | GRIN | USA |
| HamesNet Research Network | HamesNet | USA |
| Health Care for the Homeless Practice-Based Research Network | HCH PBRN | USA |
| Health Choice Network Practice Based Research Network | HCN PBRN | USA |
| Indiana University Medical Group Research Network | IUMG ResNet | USA |
| Integrated Physician Services | IPS | USA |
| Interventions to Minimize Preterm and Low Birth Weight Infants through Continuous Improvement Techniques Network | IMPLICIT | USA |
| Kansas Patients and Providers Engaged in Prevention Research | KPPEPR | USA |
| Lehigh Valley Practice Based Research Network | Lehigh Valley PBRN | USA |
| Massachusetts Academy of Family Practice Research Network | MAFP ReNet | USA |
| Medical Education Research Network | MedEdNet | USA |
| National Children's Health Project PBRN | NCHPN | USA |
| North Carolina Child Health Research Network | NCCHRN | USA |
| Northwest Ohio Primary Care Research Network | NOPCRN | USA |
| Ohio State University Primary Care Practice-Based Research Network | OSU-PCPBRN | USA |
| Penn State Ambulatory Research Network | PSARN | USA |
| Primary Care Education and Research Learning network | PEARL | USA |
| Research Association of Practices | RAP | USA |
| San Diego Unified Practice Research in Family Medicine Network | SURF*NET | USA |
| Stanford Ambulatory Research Network |  | USA |
| UCSF Collaborative Research Network | UCSFCRN | USA |
| University of California at San Francisco Collaborative Research Network | | USA |
| University of Utah Primary Care Research Network | UUPCRN | USA |
| Utah Pediatric Practice Based Research Network |  | USA |
| Virginia Ambulatory Care Outcomes Research Network | ACORN | USA |
| Washington Family Physicians Collaborative Research Network | WFPCRN | USA |
| West Virginia Practice-Based Research Network | WVPBRN | USA |
| Scripps Health PBRN | SH PBRN | USA |
| Studying, Acting, Learning, and Teaching Network | SALT-Net | USA |
| UCLA Primary Care Research Network | UCLA PCRN | USA |
| Cedars-Sinai Medical Delivery Network PBRN | Cedars-Sinai PBRN | USA |
| Cystic Fibrosis Newborn Screening Practice Based Research Network | CFNBS | USA |
| A. T. Still University, School of Osteopathic Medicine in Arizona PBRN | ATSU SOMA PBRN | USA |
| ACP Quality Connect | ACP Quality Connect | USA |
| Alabama Practice Based Research Network | APBRN | USA |
| Ambulatory Primary Care Innovations Group Network | APCIG Network | USA |
| American Association of Nurse Practitioners Network for Research | AANPNR | USA |
| Arizona Practice-Based Research Network | AzPREN | USA |
| Arkansas Research Collaborative | ARC | USA |
| Baltimore County Primary Care | BCPC | USA |
| Brigham and Women’s Primary Care Practice-Based Research Network | BWPC PBRN | USA |
| Brigham and Women's Primary Care Practice-Based Research Network | BWPC PBRN | USA |
| Building Investigative Practices for Better Health Outcomes Research Network | BIGHORN | USA |
| Cambridge Hospital Alliance Research Network | CHA Net | USA |
| Children's Hospital of Philadelphia Pediatric Research Consortium | PeRC | USA |
| Chronic Obstructive Pulmonary Disease Ventura County Medical Center PBRN | COPDVCMCPBRN | USA |
| Collaborative Partners for Primary Care Research | CPPCR | USA |
| Collaborative University Based Resources | CUBS | USA |
| Colorado Sentinel Practice Network |  | USA |
| Community Care | CC | USA |
| Community-Clinic Partnership of Los Angeles | CCPLA | USA |
| Connecticut Family Practice Obstetrics research network |  | USA |
| Consortium for Collaborative Osteopathic Research Development Practice-Based Research Network | CONCORD-PBRN | USA |
| Dayton Primary Care PBRN | DPCPBRN | USA |
| District of Columbia Primary Care Practice-Based Research Network | DC PrimCare PBRN | USA |
| East Carolina University Network | E-CARE | USA |
| Eastern Carolina Association for Research & Education | E-CARE | USA |
| Eastern Pennsylvania Inquiry Collaborative | EPICNet | USA |
| Educational networks of primary care physicians affiliated with SUNY-Upstate Medical University's Department of Family Medicine | | USA |
| Free Clinic Research & Educational Engagement Network | FREENet | USA |
| Frontier Rural Innovations Network | The Innovations Network | USA |
| Guthrie Health Care System |  | USA |
| Healthy Hearts | HH* | USA |
| Indiana Family Practice Research Network | Inet | USA |
| Institute for Family Health Research Network | IFHRN | USA |
| Integrating Medical Practice and Community-based Translational Science | IMPACTS | USA |
| Intermountain Health Care Physician Division | IHC | USA |
| International Fellowship of Primary Care Research Networks | IFPCRN | USA |
| Jacksonville Health Equity Research Organization | JaxHERO | USA |
| Johns Hopkins Community Physicians Primary Care Research Network | JHCP-PCRN | USA |
| Kansas Practice Research Network | KSPRN | USA |
| Machine On Machine Mankind Interface | M.O.M.M.I. | USA |
| Maryland Physicians Association, Inc. | MPA | USA |
| Massachusetts General Primary Care Practice Based Research Network | MGPC-PBRN | USA |
| Mecklenburg Area Partnership for Primary Care Research | MAPPR | USA |
| Meharry-Vanderbilt Community Research Network | MVCRN | USA |
| Mercer-Georgia Physician Research Network | MGPRN | USA |
| Midwifery Outcomes and Management Network | MOMNet | USA |
| Mount Sinai Practice-Based Research Network | MSPBRN | USA |
| National Nursing Center Data Mart Research Network | NNCC Data Mart | USA |
| Nemours Primary Care Research Collaborative | Nemours PCRC | USA |
| NetHaven Practice-Based Research Network |  | USA |
| New Jersey Family Physicians Network | NJFPBRN | USA |
| New York City Research & Improvement Networking Group | NYC RING | USA |
| New York-Long Island Clinical Improvement Through Innovation Collaborative | NY-LI CITI Collaborative | USA |
| North Florida Pediatric Community Research Network | NFPCRN | USA |
| Ochsner Primary Care Research Network | Ochsner-PCRN | USA |
| Ohio University Rural Ohio's Appalachian Research Network | OU-ROAR | USA |
| Ohio Valley Node | OVN | USA |
| Okinawan Remote Islands - Practice Based Research Network | Islands-PBRN | USA |
| Oklahoma Child Health Network | OCHRN | USA |
| OPTI-West Practice-Based Research Network | OPTI-WestNet | USA |
| Pediatric Diagnostic Center PBRN | PDC PBRN | USA |
| Pittsburgh Research Office Network | PRONET | USA |
| Podiatry Research Network | PRN | USA |
| Portland Research Network | PRN | USA |
| Post-Acute Therapeutics and Health (PATH) Clinical Research | PATH Clinical Research Institute | USA |
| Practice Improvement Network, a program of the Quality Improvement Innovation Networks | PIN | USA |
| Primary (Care) Practices Research Network | PPRNet | USA |
| Rainbow Research Network | RRN | USA |
| Research and Education for Academic Achievement Network | REACH Network | USA |
| Research Association of Practices of the PBRN Shared Resource | RAP | USA |
| Research Association of Practicing Physicians | RAP | USA |
| Rural Oklahoma Network | ROK-NET | USA |
| Rural Research Network of Western New York |  | USA |
| Rushmore Practice-Based Research Network | RushNet | USA |
| ShowMe Research Network | SRN | USA |
| Southeast Wisconsin Alliance for Translating Research into Practice | SWATRP | USA |
| Southern Area Patient Oriented Research Organization | SAPORO | USA |
| Southern Illinois Practice Research Organization | SIPRO | USA |
| Texas Academy of Family Physicians Research Network | TAFP R-Net | USA |
| TriValley Practice Based Research Network | TriVPBRN* | USA |
| UMass Family Medicine PBRN | UMASS-FM-PBRN | USA |
| UNC Practice Based Research Network | NCnet | USA |
| Ventura County Medical Center Diabetes Data Control Project PBRN | VCMCDDCP | USA |
| Weitzman Institute Safety Net Practice Based Research Network | WINS PBRN | USA |
| West Virginia Alliance for Creative Health Solutions | WVACHS | USA |
| West Virginia Research Network | WVRN | USA |
| Western New York Pediatric Innovation Network | WNY-PIN | USA |
